# Systems toxicology unravels multi-tiered carcinogenic networks of zearalenone in gastric cancer

**DOI:** 10.3389/fonc.2026.1762191

**Published:** 2026-03-31

**Authors:** Hangbin Zheng, Shun Chen, Guo Lin, Wenxin Huang, Yihui Guo, Hao Zhang, Ziyi Jin, Jia Lin, Yao Lin, Liwu Chen

**Affiliations:** 1 Affiliated People’s Hospital, Fujian-Macao Science and Technology Cooperation Base of Traditional Chinese Medicine-Oriented Chronic Disease Prevention and Treatment, Fujian-Hong Kong-Macau-Taiwan Collaborative Laboratory for the Inheritance and Innovation of Traditional Chinese Medicine, Fujian University of Traditional Chinese Medicine, The Academy of Rehabilitation Industry, Fujian University of Traditional Chinese Medicine, Fuzhou, Fujian, China; 2 The First School of Clinical Medicine, The Second People’s Hospital of Fujian Province, Fujian University of Traditional Chinese Medicine, Fuzhou, Fujian, China; 3 Health Medical Center, The 910th Hospital of the People’s Liberation Army Joint Logistics Support Force, Quanzhou, Fujian, China; 4 College of Integrative Medicine, Fujian University of Traditional Chinese Medicine, Fuzhou, Fujian, China; 5 The Academy of Rehabilitation Industry, Fujian University of Traditional Chinese Medicine, Fuzhou, Fujian, China

**Keywords:** gastric cancer, hormesis, machine learning, PKM2, systems toxicology, zearalenone

## Abstract

**Introduction:**

Gastric cancer (GC) is a leading cause of cancer mortality worldwide, and dietary factors like mycotoxins play a significant role in its etiology. Zearalenone (ZEN), a widespread grain contaminant, is a suspected carcinogen; however, its molecular mechanisms in GC remain unclear. This study used an integrated systems toxicology approach to identify key targets and pathways of ZEN-induced GC and validate the findings.

**Methods:**

An integrated computational and experimental strategy was used. GC-related genes were identified from Gene Expression Omnibus (GEO) datasets using differential expression and weighted gene co-expression network analysis (WGCNA). ZEN targets were obtained from multiple databases. Common targets were analyzed for pathway enrichment and protein-protein interactions. Eleven machine learning algorithms screened for core driver genes among these common targets. The binding stability of ZEN to core targets was assessed using molecular docking and 100-ns molecular dynamics simulations. In vitro functional validation was performed using CCK-8, colony formation, EdU, and wound healing assays on the human GC cell line MKN-45.

**Results:**

We identified 157 common targets of ZEN and GC. Enrichment analysis highlighted key pathways, including P13K-Akt signaling and glycolysis. Machine learning identified six core driver genes (COL1A1, INHBA, PKM2, THBS2, MFAP2, and CPA2) with high diagnostic potential (AUC>0.85). Molecular simulations confirmed ZEN forms stable complexes with core targets, particularly PKM2 and THBS2. In vitro experiments showed low concentrations of ZEN (40-80 nM) significantly promoted proliferation and migration of MKN-45 cells, demonstrating a hormetic effect.

**Discussion:**

This study suggests ZEN promotes GC progression through a multi-level network. ZEN may directly modulate key effector proteins such as PKM2 to induce metabolic reprogramming. The observed hormetic pro-proliferative and pro-migratory responses were linked to the pre-existing activation state of the PI3K/Akt pathway. These findings provide novel mechanistic insights into the carcinogenic risk of ZEN.

## Graphical Abstract

**Figure:**
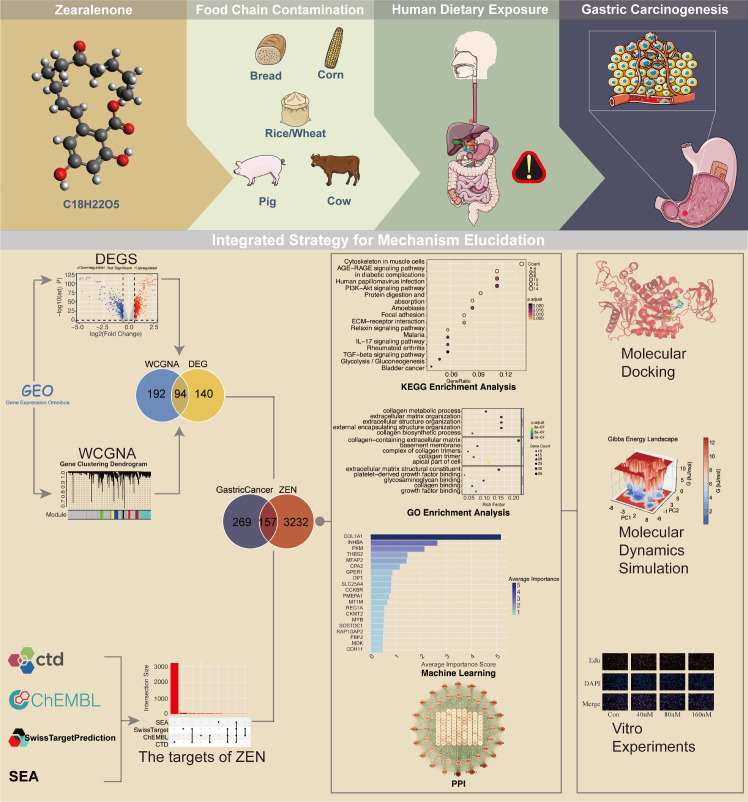
The abstract should ideally be structured according to the IMRaD format (Introduction, Methods, Results and Discussion). Provide a structured abstract if possible. If your article has been copyedited by us, please provide the updated abstract based on this version.

## 1 Introduction

The high incidence and mortality rates of gastric cancer (GC) pose a serious threat to human health worldwide. According to GLOBOCAN 2022, GC ranks fifth worldwide in terms of both cancer incidence and mortality, with approximately 968,784 new cases and 660,175 deaths (1). In addition to the significant loss of life due to its high mortality, long-term GC treatment imposes a tremendous economic burden on society. As of 2017, the cost of treating GC in China reached 23.84 billion RMB, ranking among the top three cancer-related hospitalization expenses (2). GC results from a complex interplay between the genetic background and multiple environmental risk factors. According to data from the Global Burden of Disease study, GC is one of the five major cancers most closely associated with dietary risk factors (3). Dietary habits and diet-related exposures are considered among the most critical modifiable risk factors for cancer, playing a vital role in cancer prevention and control (3).

Mycotoxins, including aflatoxins and fumonisins, which are prevalent contaminants of food crops, have garnered considerable scientific interest as a significant category of dietary contaminants with potential carcinogenic risks (4). For example, fumonisins have been linked to a high incidence of esophageal cancer, whereas aflatoxin B1 is a well-established pathogenic factor for human liver cancer (5, 6). These findings reveal a profound pathophysiological connection between certain mycotoxins and digestive-system cancers.

In this context, zearalenone (ZEN), also known as F-2 toxin, a non-steroidal estrogenic mycotoxin that contaminates grains at levels comparable to the aforementioned toxins and is also absorbed through the digestive tract, has drawn similar scrutiny (7). ZEN is primarily produced by Fusarium species, which contaminates key cereal crops and their processed products (8). Its significant thermal stability ensures persistence throughout conventional food processing, facilitating its entry into the food chain (8). A survey of over 74,000 samples from 100 countries detected ZEN in 45% of animal feed samples, with high detection rates in maize (44%), corn dried distillers grains with solubles (75%), and soybean meal (61%) (9). These high contamination levels pose a significant risk to animal feed and can be transferred through the food chain into animal-based foods, forming an indirect route of human exposure. A cross-sectional biomonitoring study of Japanese adults in 2025 found ZEN present in 71% of urine samples, indicating widespread exposure (10).

ZEN enters the human body via the contaminated food chain and is primarily metabolized in the gastrointestinal tract and liver primarily(J. 10). Prolonged exposure to ZEN has been linked to hepatotoxicity and hepatocellular carcinoma (11). However, little is known about the mechanism by which ZEN contributes to the progression of gastrointestinal tumors, such as GC. Notably, in East Asian regions, which have some of the highest global incidences of GC, ZEN contamination is prevalent in key food crops and animal feed (12–15). Whether ZEN plays a role in the occurrence and development of GC is of significant scientific and public health interest.

The pathogenesis of GC is driven by complex interactions between multiple genes and pathways. Similarly, ZEN elicits diverse biological effects through multiple molecular targets. To address this knowledge gap, we utilized network toxicology and interpretable machine learning to accurately predict the interaction networks and core driving targets of ZEN and GC using bioinformatics data. These computational predictions were validated using molecular simulations. Additionally, we performed functional experiments using human GC cell lines treated with different concentrations of ZEN. This study not only provides novel molecular evidence for the pro-carcinogenic risk of ZEN in GC but also offers an integrative research paradigm for assessing the health risks of environmental chemicals. The writing of this paper followed the TITAN 2025 rules to be clear about the use of AI in its creation (16).

## 2 Material and methods

### 2.1 Identification of differentially expressed genes

Disease targets for GC were identified using data from the Gene Expression Omnibus (GEO) database, utilizing datasets GSE29272, GSE54129, GSE27342, GSE63089, and GSE13911. For GSE27342, the exon expression matrix was transformed into a gene expression matrix using the rma function from the oligo package. The dataset selection was based on several key considerations. The integration of multiple datasets increased the sample size, enhancing statistical power and reliability. The use of microarrays with varying design principles enables technical cross-validation, reinforcing the robustness of the conclusions. The remove BatchEffect function from the limma package was used to adjust for batch effects, and the outcomes were visualized. Differential gene expression analysis was performed using the limma package. DEGs were identified using thresholds of an FDR-adjusted p-value < 0.05 and |log2FoldChange| > 1. The results were visualized as volcano plots and heatmaps using the ggplot2 package.

### 2.2 Weighted gene co-expression network analysis

To identify co-expressed gene modules in GC, we performed WGCNA using the R language WGCNA package. Through hierarchical clustering, we confirmed that there were no obvious outlier samples. To construct a topology approximating a scale-free network, we used pickSoftThreshold to select the appropriate soft-thresholding power (β), choosing the minimum power at which the scale-free topology fit index (R²) exceeded 0.85. Using blockwiseModules, we constructed the co-expression network and identified gene modules with the following parameters: networkType “unsigned”, minModuleSize 30, and mergeCutHeight 0.25. We calculated the correlations between module eigengenes (MEs) and tumor phenotype, defining core modules as those with an absolute Pearson correlation coefficient ∣r∣>0.5 and P<0.05. Scatter plots of Module Membership (MM) and Gene Significance (GS) visualized their correlation with GC phenotype.

### 2.3 Integration of GC-associated target genes

To build a robust set of GC-associated target genes, we combined DEGs with genes from WGCNA to form a unified gene set designated as GC-associated target genes.

### 2.4 Acquisition of ZEN targets

To comprehensively and systematically identify the potential targets of ZEN, we adopted an integrated strategy that combined literature-based databases with computational prediction algorithms. First, we extracted known targets supported by experimental data from two high-quality, manually curated databases, the Comparative Toxicogenomics Database (CTD) and ChEMBL, to ensure the reliability of core data. Subsequently, to explore a wider range of potential interactions, we used two mainstream prediction tools based on chemical structure similarity: SwissTargetPrediction and the Similarity Ensemble Approach (SEA). A list of potential ZEN targets was obtained by consolidating data from all four sources and removing redundancies.

### 2.5 Identification of ZEN-associated GC targets

To identify the targets of ZEN in GC, we integrated DEGs with genes identified by WGCNA as being associated with the GC phenotype to generate a set of GC-associated target genes. Concurrently, ZEN targets were consolidated from four databases: CTD, ChEMBL, SwissTargetPrediction, and SEA. Finally, the intersection of these two sets was performed to identify ZEN-associated GC targets.

### 2.6 Enrichment analysis and PPI network construction

We used the R package clusterProfiler to perform KEGG and GO pathway enrichment analyses on ZEN-associated GC targets to identify relevant biological processes. A protein-protein interaction (PPI) network was constructed using STRING. The PPI network was visualized in Cytoscape, and the MCODE plugin was applied to identify core subnetworks with the following screening parameters: Node Score Cutoff = 0.2 and K-core = 2. This analysis aimed to reveal the potential pathways within the network.

### 2.7 Machine learning and SHAP analysis

To identify core genes with predictive value for GC status from ZEN-associated targets, we divided the GEO dataset into training and validation sets using stratified sampling at a 7:3 ratio, maintaining the sample category distribution between groups. We applied Z-score normalization and added Gaussian noise to reduce the overfitting. We trained models using 11 basic algorithms (Lasso, Stepglm, plsRglm, glmBoost, Enet, Ridge, XGBoost, RF, LDA, GBM, and NaiveBayes) and 111 different configurations to construct predictive models. Using 10-fold cross-validation for hyperparameter optimization, we used the area under the receiver operating characteristic curve (AUC) as a metric for model selection. We evaluated the variable importance of each gene across the base models and ranked them according to their average importance scores to identify core driver genes. We employed the SHAP (SHapley Additive exPlanations) framework to conduct an in-depth interpretability analysis of the best-performing model. The SHAP summary plot provides a global overview of the magnitude and direction of each core gene’s contribution to model predictions. Concurrently, SHAP dependence plots were used to explore the non-linear relationships and potential interaction effects between individual gene expression levels and the model output. Finally, through SHAP force plots, we visually illustrated how the cumulative effects of the core genes drove the prediction probability from the global average baseline to its final classification outcome.

### 2.8 Molecular docking

To explore the interaction patterns between ZEN and core target proteins, we performed molecular docking. The 3D crystal structures of the target proteins were obtained from the Protein Data Bank (PDB; https://www.rcsb.org/). Proteins were pre-processed using Pymol and UCSF ChimeraX by removing water molecules, ligands, and ions and completing missing amino acid side chains. AutoDockTools was used to add polar hydrogen atoms and calculate the Gasteiger charges. The 3D structure of ZEN was retrieved from PubChem and energy-minimized using OpenBabel. Molecular docking was performed using AutoDock Vina, and the lowest binding energy conformation was selected for molecular dynamics simulations.

### 2.9 Molecular dynamics simulation

To examine the stability of the ZEN-target protein complex under physiological conditions, molecular dynamics (MD) simulations were conducted using the GROMACS software. The AMBER99SB-ILDN force field was applied to the protein, whereas the GAFF2 force field was used for ZEN. The complex system was solvated in a cubic water box using the TIP3P water model, with Na^+^ and Cl^−^ ions added for charge neutralization. The simulation followed a multi-step equilibration protocol: the system underwent 5000 steps of energy minimization using the steepest descent method. Under the NVT ensemble, the system was heated to 310 K and equilibrated for 1-ns using the V-rescale temperature coupling method. The system then transitioned to the NPT ensemble for 5-ns equilibration at 1 bar, using the Parrinello-Rahman pressure coupling method. A 100-ns MD simulation was conducted at 310 K and 1 bar, with a 2 fs integration time step. The trajectory files were analyzed to calculate root-mean-square deviation (RMSD), root-mean-square fluctuation (RMSF), and hydrogen bonds. A Gibbs Free Energy Landscape was constructed to assess conformational stability and energy distribution. The analysis results were visualized using DuIvyTools.

### 2.10 Cell culture and treatment

The human GC cell line MKN-45 was purchased from Shangen Biotechnology Co., Ltd. (Wuhan, China). Cells were cultured in 1640 medium (Gibco, USA) supplemented with 10% fetal bovine serum (FBS; Gibco, USA) and 1% penicillin-streptomycin solution (100 U/mL penicillin and 100 μg/mL streptomycin). All cells were maintained in a humidified incubator at 37 °C and 5% CO_2_. Cells in the logarithmic growth phase were used for subsequent experiments and subcultured every 2-3 days. ZEN (MCE, USA, Cat. No.: HY-103447) was dissolved in dimethyl sulfoxide (DMSO) to prepare a stock solution of ZEN. For experimental use, the stock solution was diluted to the desired concentration. Control group cells were treated with DMSO vehicle at an equivalent final concentration (<0.1%, v/v).

### 2.11 Cell counting kit-8 assay

The effect of ZEN on MKN-45 cell viability was assessed using the CCK-8 assay. Logarithmic-phase cells were seeded in 96-well plates at a density of 4×10³ cells per well. After a 24-hour incubation to allow cell adherence, the medium was replaced with fresh medium containing various concentrations of ZEN for treatment periods of 24, 48, and 72 h. At the end of each treatment period, 10 μL of CCK-8 solution (Beyotime, China, Cat. No.: C0037) was added to each well, followed by a 2-hour incubation at 37 °C, protected from light. Optical density (OD) was measured at 450 nm using a microplate reader (Bio-Rad, USA).

### 2.12 Colony formation assay

A colony formation assay was used to evaluate the role of ZEN in MKN-45 cell proliferation. The cells were digested into a single-cell suspension and seeded into 6-well plates at a density of 2,000 cells/well. After the cells adhered, they were treated with culture medium containing different concentrations of ZEN and continuously cultured in an incubator, with the drug-containing medium replaced every 3 days. The culture was terminated when visible cell colonies were observed. The cells were washed twice with PBS, fixed with 4% paraformaldehyde for 20 minutes, and then stained at room temperature with 0.1% crystal violet solution for 20 minutes. After gently rinsing with clean water and air drying, colonies with more than 50 cells were counted and photographed.

### 2.13 EdU proliferation assay

The 5-ethynyl-2’-deoxyuridine (EdU) assay kit (Beyotime, China, Cat. No.:C0075S) was used to detect cellular DNA synthesis. MKN-45 cells were seeded at a density of 1×10^5 cells per dish into 24-well plates preloaded with coverslips. After 24 hours of incubation, the cells were treated with different concentrations of ZEN for 48 hours. Subsequently, 50 μM EdU solution was added to each well, followed by incubation at 37 °C for another 2 hours. After incubation, the cells were fixed with 4% paraformaldehyde at room temperature for 15 minutes, and then permeabilized with 0.3% Triton X-100 for 30 minutes. The Click reaction was performed according to the manufacturer’s instructions. All immunofluorescence images were acquired using an inverted fluorescence microscope (Zeiss). EdU-positive cell rate (proliferation rate) = (number of EdU-positive cells/total number of DAPI-positive cells) × 100%.

### 2.14 Wound healing assay

MKN-45 cells in the logarithmic growth phase were harvested, resuspended, and seeded into 6-well plates at a density of 5×10^5^ cells per well. The cells were cultured at 37 °C in a 5% CO_2_ incubator until they formed a confluent monolayer (approximately 90–100%). Subsequently, a linear scratch was created at the center of the monolayer using a sterile 200 µL pipette tip. After washing twice with PBS to remove detached cells, the plates were cultured in 1640 medium containing different concentrations of ZEN. The control group received medium with an equivalent amount of DMSO (final concentration <0.1%). Images of the scratch wound at the same pre-marked locations were captured at 0 and 48 hours. The wound area at each time point was measured using the ImageJ software. The lateral migration ability of the cells was quantitatively assessed by calculating the wound healing rate with the formula: Wound Healing Rate (%) = [(Area at 0h − Area at 48h)/Area at 0h] × 100%.

### 2.15 Statistical analysis

All *in vitro* experiments were independently repeated three times (n=3). The experimental data were analyzed and visualized using GraphPad Prism software. Quantitative data are presented as mean ± standard deviation (mean ± SD). Comparisons among multiple groups were conducted using one-way analysis of variance (ANOVA), followed by Tukey’s multiple comparisons *post hoc* test for pairwise comparisons. All statistical tests were two-sided, and a P-value < 0.05 was considered to indicate a statistically significant difference.

## 3 Results

### 3.1 Identification of differentially expressed genes in GC

**Figure 1:**
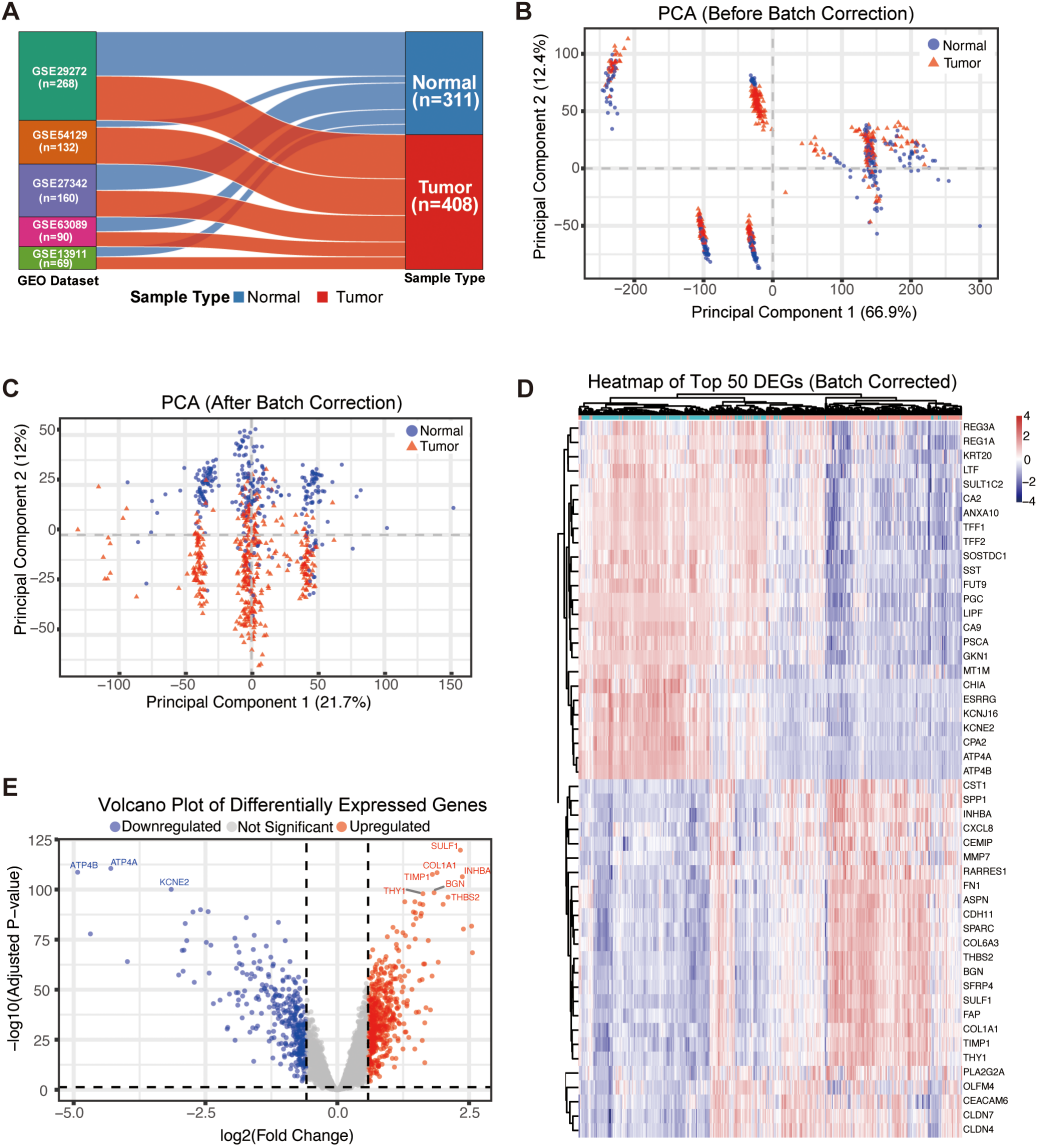
Identification and analysis of differentially expressed genes (DEGs) in GC. **(A)** Bar chart illustrating the sample composition from five GEO datasets, totaling 408 tumor samples and 311 normal samples. **(B)** Principal Component Analysis (PCA) plot of samples before batch effect correction, showing distinct clustering by dataset. **(C)** PCA plot after batch effect correction using the ComBat algorithm, demonstrating the successful removal of batch effects. **(D)** Heatmap of the top 50 most significant DEGs, showing distinct expression profiles that clearly differentiate between tumor (red) and normal (blue) samples. **(E)** Volcano plot displaying all DEGs between tumor and normal tissues. Upregulated genes are shown in red, downregulated genes in blue, and non-significant genes in gray. Genes were screened based on an FDR-adjusted p-value < 0.05 and |log2(Fold Change)| > 1.

We analyzed a total of 311 normal samples and 408 tumor samples (**Figure 1A**). Principal component analysis (PCA) revealed that prior to batch correction, samples were clustered predominantly by dataset origin, indicating significant batch effects (**Figure 1B**). After applying the ComBat algorithm, batch effects were eliminated (**Figure 1C**). Differential expression analysis identified a total of 234 DEGs (**Supplementary Table 1**), including 114 upregulated and 120 downregulated genes, as shown in the volcano plot (**Figure 1E**). To validate the expression patterns of these DEGs and their ability to distinguish between sample types, we generated a heatmap of the 50 most significant DEGs. The expression profiles of the DEGs clearly differentiated tumor samples from normal samples (**Figure 1D**).

### 3.2 Discovery of core GC modules via weighted gene co-expression network analysis

**Figure 2:**
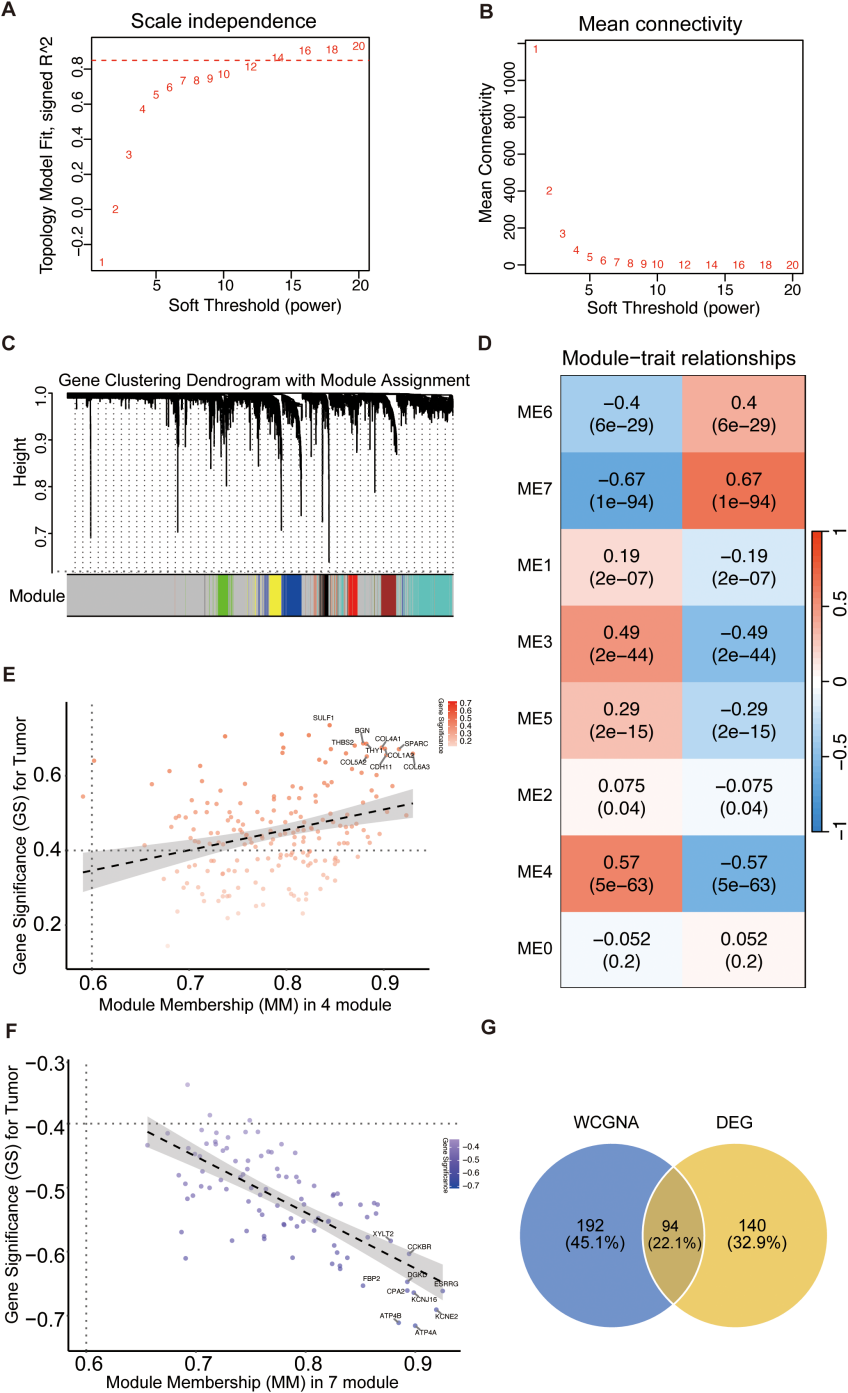
Identification of key GC-associated gene modules via WGCNA. **(A)** Analysis of the scale-free fit index for various soft-thresholding powers (β). **(B)** Analysis of the mean connectivity for various soft-thresholding powers. A power of β=14 was selected to ensure a scale-free network topology. **(C)** Dendrogram of gene clustering with corresponding module assignments indicated by color. Seven distinct co-expression modules were identified in this study. **(D)** Heatmap of module-trait relationships showing the correlation between module eigengenes and tumor status. The ME4 (positively correlated) and ME7 (negatively correlated) modules were identified as the most significant modules. **(E, F)** Scatter plots showing the correlation between Gene Significance **(GS)** for tumor status and Module Membership (MM) in the ME4 **(E)** and ME7 **(F)** modules, confirming their biological relevance. **(G)** Venn diagram illustrating the integration of 286 genes from the core WGCNA modules and 234 DEGs, resulting in a final set of 426 GC-associated target genes.

We employed WGCNA to identify the key gene modules associated with GC. A soft-thresholding power of β = 14 was selected to construct a scale-free network (**Figures 2A, B**). Subsequently, the dynamic tree cut algorithm was used to detect seven co-expression modules (**Figure 2C**; **Supplementary Table 2**). By calculating the correlations between module eigengenes (ME) and tumor status, we determined that the ME4 module, comprising 191 genes, and the ME7 module, consisting of 95 genes, exhibited the most significant positive and negative associations with the GC phenotype, respectively (**Figure 2D**). The strong concordance between gene significance (GS) and module membership (MM) within each module further corroborated the central biological roles of these two modules (**Figures 2E, F**). Consequently, we designated ME4 and ME7 as core modules, yielding 286 key genes influencing GC progression for further analysis. The integration of these WGCNA-derived genes with DEGs resulted in a final GC-associated target gene set of 426 genes (**Figure 2G**).

### 3.3 Identification and enrichment analysis of ZEN-associated GC targets

**Figure 3:**
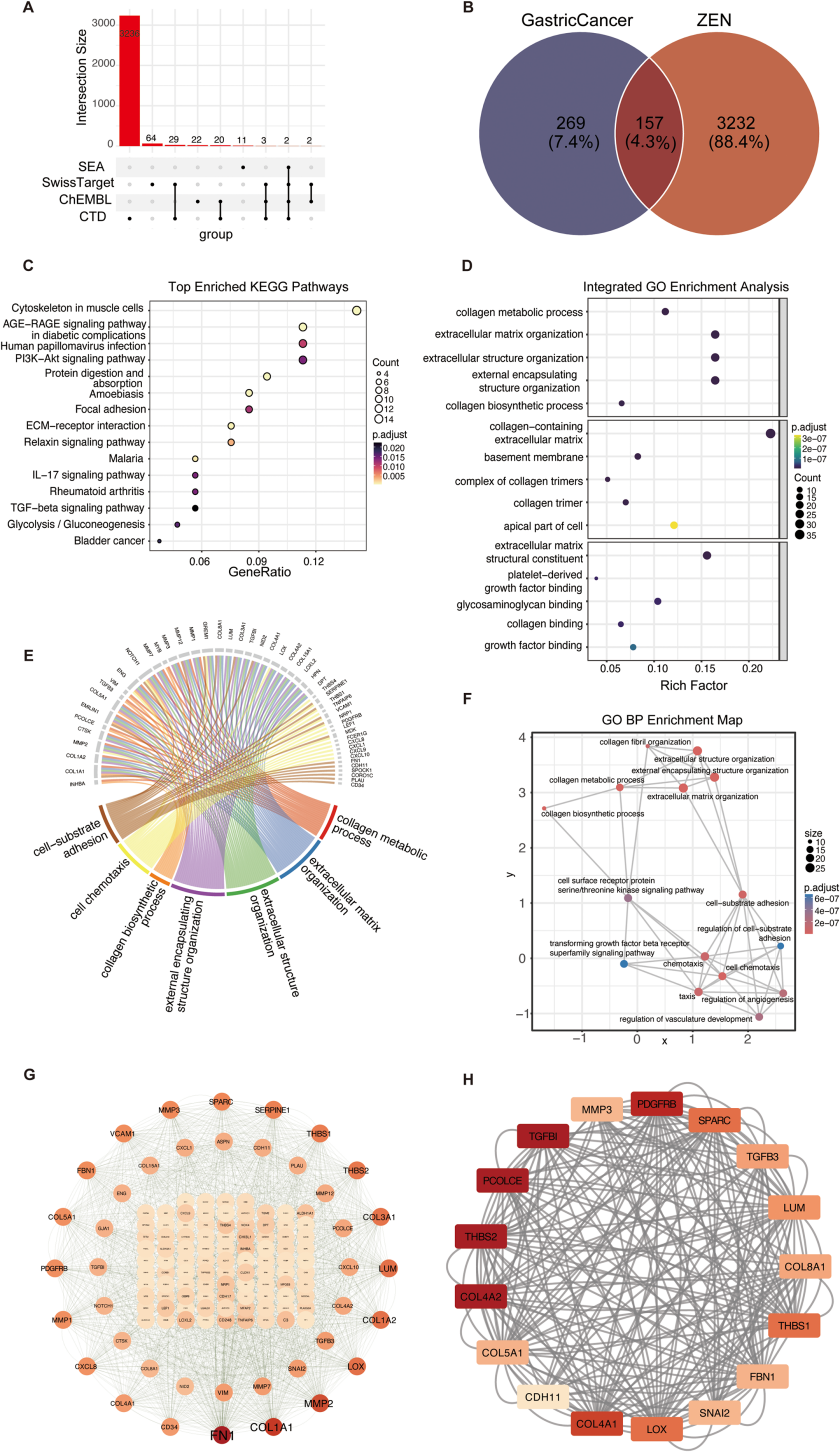
Identification and functional enrichment analysis of ZEN-associated GC targets. **(A)** Upset plot showing the distribution of 3,389 potential ZEN targets identified from CTD, ChEMBL, SwissTargetPrediction, and SEA databases. **(B)** Venn diagram showing the intersection of 3,389 ZEN-related targets and 426 GC-associated genes, yielding 157 common targets. **(C)** Dot plot of the top enriched KEGG pathways for the 157 common targets. **(D)** Dot plot of the top enriched Gene Ontology (GO) terms, categorized by Biological Process (BP), Cellular Component (CC), and Molecular Function (MF). **(E)** Chord diagram illustrating the relationship between the core target genes and enriched KEGG pathways. **(F)** GO BP enrichment map showing functional clustering of enriched processes related to extracellular matrix remodeling and cell adhesion. **(G)** Protein-Protein Interaction **(PPI)** network of the 157 core targets. Node color corresponds to the degree of connectivity. **(H)** The most significant functional module identified from the PPI network using the MCODE plugin, containing 17 key genes.

The integration of the CTD, ChEMBL, SwissTargetPrediction, and SEA databases identified 3389 potential ZEN targets (**Figure 3A**; **Supplementary Table 3**). An intersection analysis between these targets and 426 GC-associated target genes revealed 157 overlapping genes (**Figure 3B**; **Supplementary Table 4**), which were defined as ZEN-associated GC targets. Functional enrichment analyses of these targets showed significant enrichment in cancer-related signaling pathways (**Figure 3C**; **Supplementary Table 5**), including PI3K-Akt signaling, extracellular matrix (ECM)-receptor interaction, focal adhesion, and glycolysis/gluconeogenesis, which regulated cell proliferation, invasion, and metastasis. GO enrichment analysis (**Figure 3D**) showed that the 157 targets were involved in extracellular matrix organization and cell-substrate adhesion (Biological Process), located in collagen trimer and extracellular matrix (Cellular Component), and associated with extracellular matrix structural constituent and integrin binding (Molecular Function). The gene-pathway crosstalk diagram demonstrated the pleiotropic roles of the 157 targets across multiple pathways (**Figure 3E**). An enrichment map of GO biological processes revealed that the enriched processes clustered into a functional group related to extracellular matrix remodeling, cell adhesion, and angiogenesis regulation, highlighting ZEN’s key influence on GC (**Figure 3F**). The protein-protein interaction network of the 157 targets and subsequent MCODE analysis identified a core functional module containing 17 genes critical for ECM remodeling and tumor invasion/metastasis (**Figures 3G, H**).

### 3.4 Core genes screening based on machine learning and SHAP analysis

**Figure 4:**
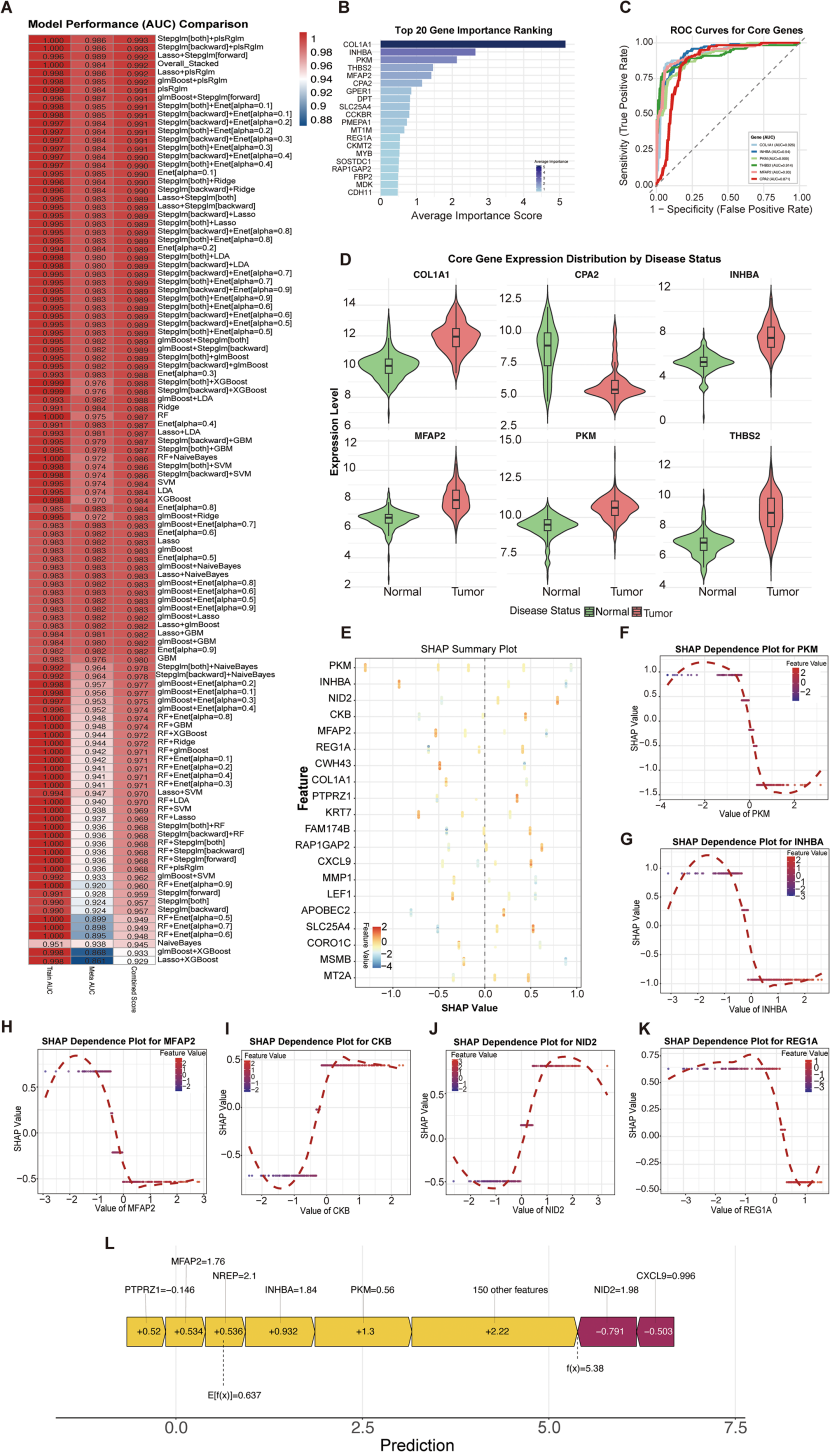
Machine learning-based screening and interpretability analysis of core driver genes. **(A)** Heatmap comparing the Area Under the Curve **(AUC)** performance of 111 machine learning models. The Stepglm[both]+plsRglm model achieved the best performance. **(B)** Bar chart ranking the top 20 genes by their average importance score across all models. **(C)** Receiver Operating Characteristic (ROC) curves for the six core genes (COL1A1, INHBA, PKM, THBS2, MFAP2, CPA2), demonstrating their robust standalone classification capability (AUC > 0.85). **(D)** Violin plots showing the expression distribution of the six core genes in tumor versus normal tissues. **(E)** SHAP summary plot illustrating the global impact and direction of the top features on the model’s predictions. **(F-K)** SHAP dependence plots showing the non-linear relationship between the expression of individual core genes and their corresponding SHAP values. **(L)** SHAP force plot for a single prediction, illustrating the cumulative and antagonistic contributions of multiple genes driving the final classification outcome.

To identify core genes from the 157 potential targets, we systematically evaluated 111 models using 11 machine learning algorithms. Analysis showed that the Stepglm[both]+plsRglm model exhibited superior classification performance (**Figure 4A**; **Supplementary Table 6**). Among the top 20 genes ranked by average importance score (**Figure 4B**), *COL1A1, INHBA, PKM, THBS2, MFAP2*, and *CPA2* had scores exceeding 1 (**Supplementary Table 7**), indicating their pivotal role in the model prediction. To assess the independent predictive value of these six genes, we evaluated their classification efficacy as standalone predictors. ROC curve analysis confirmed AUC values above 0.85 for each gene, demonstrating a robust standalone classification capability (**Figure 4C**). Visualization of their expression distribution revealed significantly elevated levels in tumor tissues compared to normal tissues for all genes except *CPA2*, providing transcriptomic evidence of their oncogenic roles (**Figure 4D**). The SHAP summary plot (**Figure 4E**, **Supplementary Table 8**) provides global insights and shows the impact direction of the predictions. High expression of *PKM, INHBA, MFAP2, CWH43*, and *COL1A1* positively drove SHAP values, suggesting an increased GC risk; conversely, high *NID2* and *CKB* expression showed inhibitory effects. SHAP dependence plots elucidated the non-linear relationship between these genes and model predictions (**Figures 4F–K**). The global SHAP force plot shows the decision-making process of the model (**Figure 4L**), illustrating that the final predictive score was achieved through an antagonistic and cumulative balance between promoting genes (*PKM*, *INHBA*, *NREP*, *MFAP2*, *and PTPRZ1*) and inhibiting genes (*NID2* and *CXCL9*). By integrating evidence from model contribution, predictive performance, expression patterns, and SHAP contribution direction, we identified *COL1A1*, *INHBA*, *PKM*, *THBS2*, *MFAP2*, and *CPA2* as the core target genes for ZEN-induced GC.

### 3.5 Molecular docking reveals ZEN-target interactions

**Figure 5:**
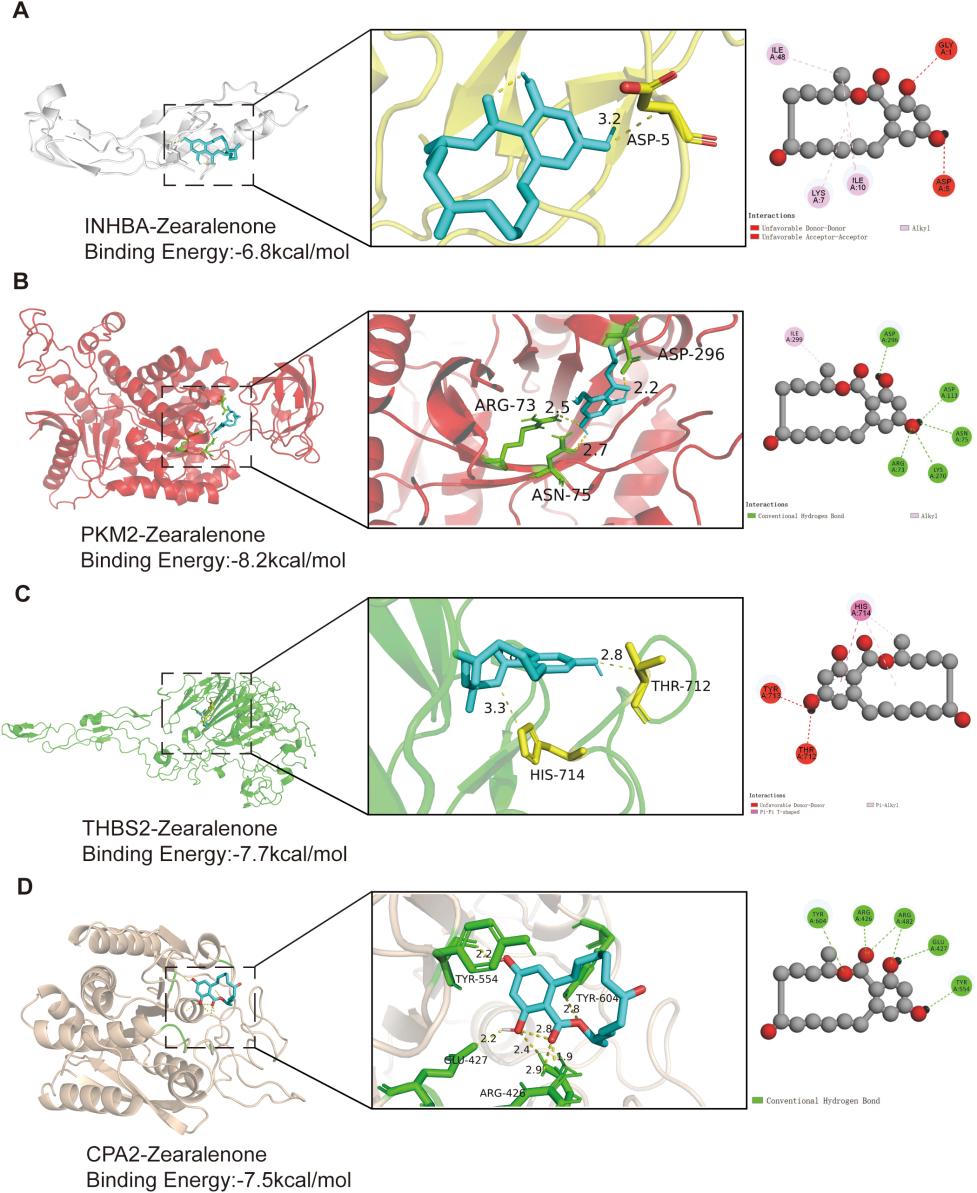
Molecular docking analysis of ZEN with core target proteins. 3D and 2D diagrams illustrating the binding conformations and intermolecular interactions between ZEN and four core target proteins. The binding energy for each complex is provided. **(A)** INHBA-ZEN complex (Binding Energy: -6.8 kcal/mol). **(B)** PKM2-ZEN complex (Binding Energy: -8.2 kcal/mol). **(C)** THBS2-ZEN complex (Binding Energy: -7.7 kcal/mol). **(D)** CPA2-ZEN complex (Binding Energy: -7.5 kcal/mol).

We conducted molecular docking analyses to investigate the binding ability of ZEN to the core target proteins INHBA (PDB ID: 2ARP), PKM2 (PDB ID: 8G2E), THBS2 (PDB ID: 1YO8), and CPA2 (PDB ID: 1DTD) (**Figure 5**). The binding energies of ZEN with PKM2, THBS2, and CPA2 were below -7.0 kcal/mol, suggesting that ZEN had strong binding activity with these proteins. PKM2 exhibited the lowest binding energy (-8.2 kcal/mol), indicating that it may serve as the main target for physical interactions. Analysis of the binding conformations showed that complex stability was dominated by different intermolecular interactions. The stability of ZEN and INHBA was attributed to hydrophobic alkyl interactions between ZEN and the ILE and LYS residues of INHBA, although repulsive interactions with GLY and ASP residues partially weakened binding (**Figure 5A**). The high stability between ZEN and PKM2 stemmed from a hydrogen-bond network formed by the hydroxyl groups of ZEN and the ASP, ASN, LYS, and ARG residues in the PKM2 active pocket (**Figure 5B**). The binding of ZEN to THBS2 depends on nonpolar interactions, particularly π–π T-shaped stacking between the benzene ring of ZEN and the TYR residue of THBS2. The π system of ZEN formed Pi-Alkyl interactions with the HIS residue of THBS2, anchoring the ligand in the binding pocket (**Figure 5C**). The binding between ZEN and CPA2 was driven by six conventional hydrogen bonds between the functional groups of ZEN and TYR, ARG, and GLU residues in CPA2, which created a strong binding activity (**Figure 5D**). These results demonstrate the capacity of ZEN to securely embed within the active pockets of these four core targets, providing a structural basis for subsequent functional effects.

### 3.6 Molecular dynamics validate complex stability

**Figure 6:**
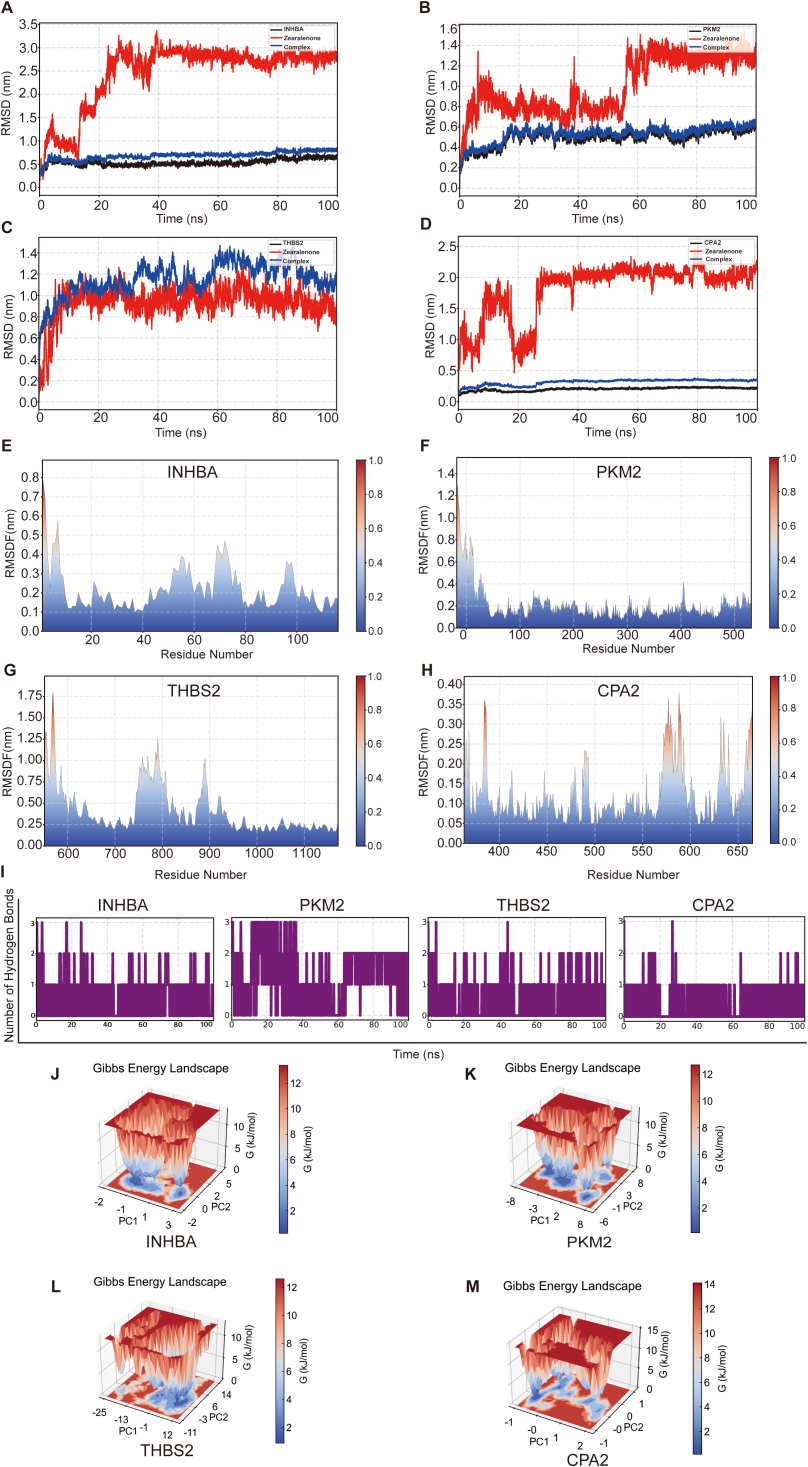
Molecular dynamics (MD) simulation of ZEN-protein complexes over 100 ns. **(A-D)** Root Mean Square Deviation (RMSD) plots for the protein backbone (black), ligand ZEN (red), and the entire complex (blue) for INHBA **(A)**, PKM2 **(B)**, THBS2 **(C)**, and CPA2 **(D)**. **(E-H)** Root Mean Square Fluctuation (RMSF) plots for each residue of INHBA **(E)**, PKM2 **(F)**, THBS2 **(G)**, and CPA2 **(H)**, indicating local protein flexibility. **(I)** Plot showing the number of hydrogen bonds maintained between ZEN and each of the four target proteins throughout the simulation. **(J-M)** Gibbs free energy landscape maps for the INHBA **(J)**, PKM2 **(K)**, THBS2 **(L)**, and CPA2 **(M)** complexes, illustrating conformational stability and energy distribution. Deeper blue wells indicate more stable conformations.

To elucidate the dynamic behavior and stability of the complexes formed by ZEN and the four core proteins (INHBA, PKM2, THBS2, and CPA2), we conducted 100 ns molecular dynamics simulations. Root mean square deviation (RMSD) analysis was used to assess the overall structural stability. The RMSD values for the INHBA protein backbone and the entire complex rapidly stabilized at approximately 0.5 nm at the onset of the simulation and remained constant, indicating the preservation of the folded structure (**Figure 6A**). However, the RMSD of ligand ZEN shifted after 40 ns and stabilized at approximately 2.5 nm, suggesting that a conformational transition occurred in the binding pocket, ultimately resulting in a more stable binding state. The PKM2-ZEN complex and its protein backbone also exhibited good structural stability (**Figure 6B**). The ligand ZEN underwent a notable conformational shift at approximately 55 ns (RMSD increased from ~0.8 nm to ~1.3 nm), reflecting an adjustment of its position within the binding pocket. In contrast, all components of the THBS2-ZEN complex exhibited exceptional stability, with RMSD values fluctuating minimally (~0.2 nm) throughout the simulation, demonstrating the formation of a highly rigid system (**Figure 6C**). The protein backbone and the overall CPA2 complex were also extremely stable (RMSD fluctuation ~0.2 nm), but the RMSD of the ligand ZEN showed a sharp transition at approximately 25 ns, indicating a high degree of instability and suggesting that the ligand underwent a conformational rearrangement from its initial binding pocket (**Figure 6D**).

Root mean square fluctuation (RMSF) analysis was used to explore local protein flexibility. The RMSF analysis revealed that most regions of INHBA were notably rigid (RMSF < 0.2 nm), although a highly flexible loop region was identified around residues 50-80 (**Figure 6E**). PKM2 exhibited the greatest overall rigidity, with most residues showing fluctuation values below 0.2 nm, indicating an exceptionally stable three-dimensional backbone (**Figure 6F**). THBS2 displayed strong rigidity in most areas but had a region near residue 800, where the fluctuation peak exceeded 1.0 nm, suggesting the presence of an extremely flexible, long-chain structure (**Figure 6G**). CPA2’s overall rigidity was similar to that of PKM2 (RMSF < 0.3 nm), with its main flexible region located near the C-terminus (**Figure 6H**).

The analysis of hydrogen bonds evaluated the stability of the key polar interactions (**Figure 6I**). INHBA and ZEN maintained 1-3 hydrogen bonds before 40 ns, predominantly stabilizing at one bond thereafter. PKM2 and ZEN formed the most persistent and stable hydrogen bond network, consistently maintaining three hydrogen bonds during the initial 40 ns, and for the remainder of the simulation, the number of hydrogen bonds mostly remained at two, indicating a highly stable binding mode. The number of hydrogen bonds between THBS2 and ZEN exhibited dynamic fluctuations, varying from one to two. Hydrogen bonding between CPA2 and ZEN was the weakest, with only one hydrogen bond maintained during most of the simulation period. Overall, although the ligand in all systems was capable of interacting with the protein, the PKM2-ZEN complex demonstrated the most stable polar interactions, while the hydrogen bonds in the other three systems displayed more dynamic.

Gibbs free energy landscape maps explore the energy states of conformational space. The energy landscapes of INHBA and THBS2 both featured deep and concentrated low-energy wells, indicating that the complexes formed between THBS2 or INHBA and the ligand were highly stable (**Figures 6J, L**). PKM2 displayed at least two independent energy wells connected by a low-energy barrier, suggesting that the PKM2-ZEN complex is dynamically flexible and capable of transitioning between different stable conformational states (**Figure 6K**). The energy landscape of CPA2 contained several scattered shallow energy wells and lacked a distinct energy minimum, further confirming the conformational instability and relatively weak binding affinity of the CPA2-ZEN complex (**Figure 6M**).

### 3.7 ZEN promotes the proliferation and migration of MKN45 cells

**Figure 7:**
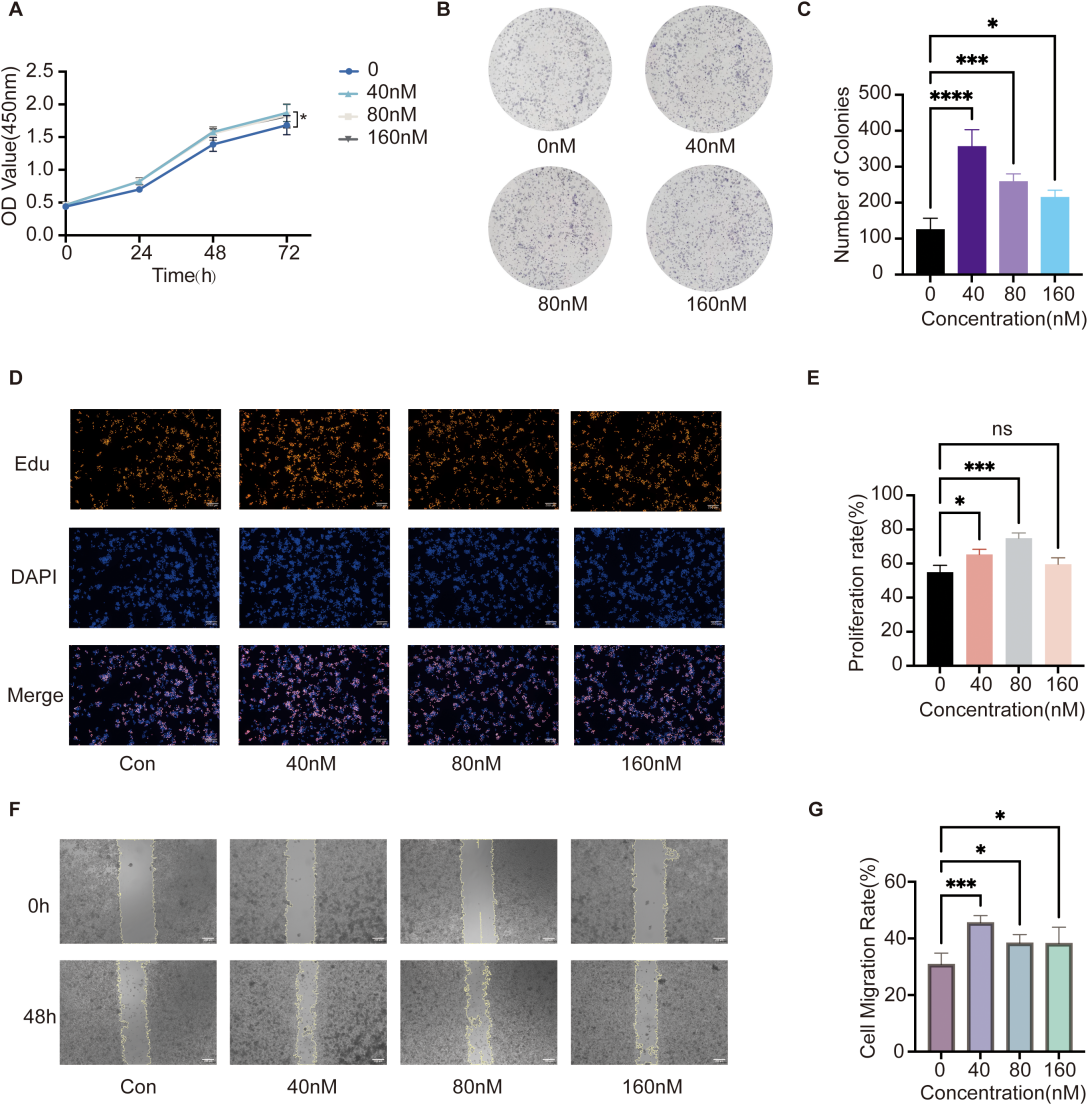
*In vitro* validation of ZEN’s effects on the proliferation and migration of MKN-45 GC cells. **(A)** CCK-8 assay results showing the viability of MKN-45 cells after treatment with different concentrations of ZEN (0, 40, 80 and 160 nM) for 24, 48, and 72 hours. **(B)** Representative images of the colony formation assay after 14 days of treatment with ZEN. **(C)** Quantification of the number of colonies formed. **(D)** Representative immunofluorescence images from the EdU proliferation assay. EdU-positive (proliferating) cells are shown in red, and DAPI-stained nuclei are in blue. **(E)** Quantification of the EdU proliferation rate. **(F)** Representative images of the wound healing assay at 0 and 48 hours after treatment with ZEN. **(G)** Quantification of the cell migration rate. Data are presented as mean ± SD from three independent experiments. *P < 0.05, ***P < 0.001, ****P < 0.0001, ns, not significant.

We assessed the effects of ZEN on the proliferation and migration of the human GC cell line MKN-45. The CCK-8 assay demonstrated that ZEN significantly enhanced the proliferation of MKN-45 cells, although this effect was non-linear and dose-dependent (**Figure 7A**). The colony formation assay revealed that all concentrations of ZEN (40, 80, and 160 nM) markedly increased the colony-forming capacity of the cells (P<0.05). Notably, the proliferative effect was most pronounced at 40 nM, while 80 nM and 160 nM also exhibited significant promotive effects (P<0.05) (**Figures 7B, C**). The EdU proliferation assay further corroborated the proliferative effect at lower concentrations, with findings indicating that both 40 nM and 80 nM ZEN significantly elevated the DNA synthesis rate in the cells (**Figures 7D, E**). Regarding cell migration, after 48 hours of treatment with varying concentrations of ZEN, all groups demonstrated significantly accelerated scratch wound closure, suggesting enhanced cell migration capability (**Figures 7F, G**). Collectively, these results indicate that ZEN can significantly promote the proliferation and migration of MKN-45 cells *in vitro*, thereby functionally confirming its potential pro-carcinogenic effects.

## 4 Discussion

Gastric Cancer is a widespread malignant tumor that results from interactions between genetic predispositions and environmental factors. Among dietary risk factors, the molecular mechanisms by which the mycotoxin ZEN, a widespread grain contaminant, contributes to gastric carcinogenesis remain incompletely characterized. This study employed an integrative approach combining network toxicology, interpretable machine learning, and experimental validation to identify the shared molecular targets and key regulatory pathways linking ZEN exposure to GC progression. Critically, *in vitro* functional assays demonstrated that low-dose ZEN exposure significantly enhanced the proliferation and migration of human GC MKN-45 cells.

KEGG enrichment analysis clearly pointed to the glycolysis/gluconeogenesis pathway, and interpretable machine learning prioritized PKM2 as a core driver gene. PKM2 is frequently overexpressed in GC and acts as a key regulatory factor for the proliferation of GC cells, supporting cell growth by maintaining high levels of aerobic glycolysis(H. 17). As the central enzyme regulating the Warburg effect, PKM2 precisely balances the energetic output of glycolysis and the supply of biosynthetic precursors by switching between its highly active tetrameric and less active dimeric forms, thereby supporting rapid proliferation of tumors (18). Molecular dynamics simulations demonstrated ZEN’s stable occupation of a specific binding pocket within PKM2, maintained through a robust hydrogen-bond network. We speculate that this binding may allosterically stabilize PKM2 in its hyperactive tetrameric conformation(T.-J. 19), thereby perpetuating glycolytic flux to provide energy and macromolecular precursors for uncontrolled proliferation, consistent with our observed pro-proliferative phenotypes.

The proteins COL1A1, INHBA, THBS2, and MFAP2 identified in our study are integral components of ECM-receptor interactions and cell migration. As ECM constituents or paracrine factors, these proteins facilitated cell migration and invasion in GC through multiple signaling pathways. Our wound healing assays demonstrated that ZEN significantly accelerated the migration of MKN-45 cells, which functionally validates the roles of these structural effectors. For instance, Research showed that transcription factor RUNX2 enhances migration, invasion, and *in vivo* metastasis of GC cells by upregulating ECM protein COL1A1(20). Similarly, THBS2 promotes proliferation, migration, invasion, epithelial-mesenchymal transition (EMT), and cancer stem cell characteristics in GC cells by activating the Notch signaling pathway, contributing to tumor progression and metastasis (21). Notably, a recent study in gastrointestinal cancers has elucidated a novel mechanism where THBS2 mediates the activation of the PI3K/AKT signaling pathway via the COL8A1-ITGB1 axis, thereby promoting EMT and progression (22). This aligns with our systems toxicology prediction and *in vitro* validation. Specifically, the observed promotion of MKN-45 migration could be attributed to this THBS2-driven PI3K/AKT activation, which serves as a multi-tiered regulatory network alongside the PKM2-mediated metabolic reprogramming. In molecular dynamics simulations, the THBS2-ZEN complex showed structural stability, providing a molecular explanation for ZEN’s role in promoting migration. INHBA, secreted by FAP+ GC mesenchymal stromal cells, enhances cancer cell proliferation and migration, serving as a pro-oncogenic paracrine factor (23). MFAP2 augments GC cell motility and is highly expressed in GC tissues, promoting cell motility and EMT by activating integrin/FAK/ERK and TGF-β/SMAD pathways (24). Studies showed ZEN promoted migration and invasion in endometrial cancer cells by activating estrogen receptor-α, triggering Rho/ROCK/PMLC signaling, leading to cytoskeletal remodeling, reduced cell adhesion, and EMT induction (25).Interestingly, the subnetwork components identified by our MCODE analysis were also closely related to ECM remodeling and metastasis in cancer cells. These findings provide a molecular basis for our observation that ZEN enhances MKN-45 the migration capacity. CPA2 was identified as a core driver owing to its high diagnostic efficacy, highlighting a novel candidate largely unexplored in gastric cancer. Despite its established prognostic role in pancreatic cancer (26), the conformational instability and shallow energy landscape observed in MD simulations suggest CPA2 serves as a secondary effector within the multi-tiered ZEN-GC network compared to more stable interactions like PKM2.

Notably, the PI3K/Akt signaling pathway, highlighted in the enrichment results, plays a central role in the initiation, progression, and metastasis of GC (27). During GC progression, the PI3K/Akt pathway acts as a critical oncogenic hub, persistently activated by genetic mutations, pathogenic infections, and various upstream signals, such as growth factors (27). This signaling pathway facilitates the uncontrolled proliferation of tumor cells by inhibiting cell cycle suppressors (28). In terms of invasion and metastasis, the PI3K/Akt pathway works synergistically with signals such as TGF-β and plays a pivotal role in the EMT process of cancer cells (29). Furthermore, the PI3K/Akt pathway not only regulates glycolysis (30) but also serves as a crucial hub for ECM remodeling (31–33).

Previous reports have indicated that ZEN exerts bidirectional effects on PI3K/Akt signaling depending on the cell type and exposure dose, potentially explaining the hormesis phenomenon observed in our *in vitro* studies (34–36). Hormesis is a widespread adaptive response in various organisms and stressors, characterized by a non-linear biphasic dose–response relationship (37). Our *in vitro* experiments demonstrated that ZEN exerted a complex dose-dependent effect on GC cells, significantly promoting proliferation and migration at low concentrations (40 – 80 nM), while the promoting effect diminished at higher concentrations. This non-monotonic dose-response relationship aligns with established hormetic patterns, wherein low-dose stimulation transitions to high-dose inhibition, an evolutionarily conserved adaptive response potentially mediated through PI3K/Akt pathway modulation (38). Therefore, the hormesis effect of ZEN challenges the traditional toxicological assumption that “the dose makes the poison”, suggesting that current food safety standards based on high-dose data may not adequately protect against the cancer-promoting risks of long-term, low-dose exposure. This underscores the need for refined risk assessment frameworks that incorporate non-linear dose-response relationships in environmental carcinogenesis.

## 5 Conclusion

This study systematically elucidated the mechanisms through which ZEN induces GC by combining computational predictions with experimental validation. Drawing from multidimensional evidence, we consequently propose a central hypothesis: the pro-proliferative and pro-migratory effects of ZEN on GC cells stem from a multi-level, synergistically regulated network rather than a single pathway. Specifically, this network functions on two levels: ZEN may directly modulate core effector proteins, such as PKM2 and THBS2, to drive metabolic reprogramming and extracellular matrix remodeling, while concurrent dose-dependent modulation of the PI3K/Akt pathway underlies the hormetic responses observed at low ZEN concentrations *in vitro*. While this hypothesis offers a comprehensive perspective for understanding the carcinogenic mechanisms of ZEN, certain refinements remain necessary. For instance, although MKN-45 served as a representative model, future studies incorporating diverse cell lines will be essential to further generalize these findings. Furthermore, subsequent research should focus on verifying the direct allosteric effects of ZEN on PKM2 and clarifying the role of the PI3K/Akt signaling pathway in ZEN-induced hormesis. Ultimately, expanding research to the epidemiological level could help explore the association between ZEN exposure and human GC risk. Such research would substantiate our proposed mechanisms and inform evidence-based public health policies.

## Data Availability

The datasets presented in this study can be found in online repositories. The names of the repository/repositories and accession number(s) can be found in the article/supplementary material.

## Glossary

ANOVA: Analysis of Variance
AUC: Area Under the Receiver Operating Characteristic Curve
BP: Biological Process
CC: Cellular Component
CCK-8: Cell Counting Kit-8
CKB: Creatine Kinase B
COL1A1: Collagen Type I Alpha 1 Chain
CPA2: Carboxypeptidase A2
CTD: Comparative Toxicogenomics Database
CWH43: Cell Wall Biogenesis 43 C-Terminal Homolog
CXCL9: C-X-C Motif Chemokine Ligand 9
DAPI: 4’,6-diamidino-2-phenylindole
DEGs: Differentially Expressed Genes
DMSO: Dimethyl Sulfoxide
ECM: Extracellular Matrix
EdU: 5-ethynyl-2’-deoxyuridine
Enet: Elastic Net
EMT: Epithelial-Mesenchymal Transition
ERK: Extracellular Signal-Regulated Kinase
FAK: Focal Adhesion Kinase
FAP: Fibroblast Activation Protein
FBS: Fetal Bovine Serum
FDR: False Discovery Rate
GBM: Gradient Boosting Machine
GC: Gastric Cancer
GEO: Gene Expression Omnibus
glmBoost: Gradient Boosting with Regression Trees
GO: Gene Ontology
GS: Gene Significance
INHBA: Inhibin Subunit Beta A
KEGG: Kyoto Encyclopedia of Genes and Genomes
Lasso: Least Absolute Shrinkage and Selection Operator
LDA: Linear Discriminant Analysis
MD: Molecular Dynamics
Me: Module Eigengenes
MF: Molecular Function
MFAP2: Microfibril Associated Protein 2
MM: Module Membership
NaiveBayes: Naive Bayes
NID2: Nidogen 2
NPT: Isothermal-Isobaric Ensemble
NREP: Neuronal Regeneration Related Protein
NVT: Canonical Ensemble
OD: Optical Density
PCA: Principal Component Analysis
PDB: Protein Data Bank
PI3K-Akt: Phosphoinositide 3-kinase/Akt
PKM2: Pyruvate Kinase M2
plsRglm: Partial Least Squares Logistic Regression
PMLC: Phosphorylated Myosin Light Chain
PPI: Protein-Protein Interaction
PTPRZ1: Receptor Type Protein Tyrosine Phosphatase Zeta 1
RF: Random Forest
Ridge: Ridge Regression
Rho/ROCK: Ras homolog family member A/Rho-associated coiled-coil containing protein kinase
RMSD: Root-Mean-Square Deviation
RMSF: Root-Mean-Square Fluctuation
ROC: Receiver Operating Characteristic
RUNX2: Runt-related transcription factor 2
SD: Standard Deviation
SEA: Similarity Ensemble Approach
SHAP: SHapley Additive exPlanations
SMAD: SMAD family member
Stepglm: Stepwise Generalized Linear Model
TGF-β: Transforming Growth Factor-β
THBS2: Thrombospondin 2
WGCNA: Weighted Gene Co-expression Network Analysis
XGBoost: Extreme Gradient Boosting
ZEN: Zearalenone
